# Association Between Serum Vitamin D Levels and Neck of Femur Fractures in the Elderly: A Cross-Sectional Observational Study

**DOI:** 10.7759/cureus.94199

**Published:** 2025-10-09

**Authors:** Akshay K Sharma, Anil K Juyal, Harsh Priyadarshi

**Affiliations:** 1 Department of Orthopedics, Himalayan Institute of Medical Sciences, Dehradun, IND

**Keywords:** bone health, elderly, fragility fracture, neck of femur fracture, osteoporosis, risk factors, vitamin d deficiency

## Abstract

Background: Neck of femur fractures represent a major public health concern in the elderly, often leading to significant morbidity, mortality, and functional impairment. Vitamin D deficiency (VDD), a modifiable risk factor, is increasingly recognized for its role in bone fragility and fracture susceptibility in older adults.

Objectives: The present study aimed to assess the association between serum vitamin D levels and neck of femur fractures in elderly patients and identify demographic and clinical variables influencing this relationship.

Methods: This cross-sectional observational study was conducted over 18 months at the Department of Orthopedics, Himalayan Institute of Medical Sciences, India. A total of 116 patients aged ≥60 years with radiologically confirmed neck of femur fractures were enrolled. Serum 25-hydroxyvitamin D (25(OH)D) levels were measured using enzyme-linked immunosorbent assay (ELISA) and categorized as normal, insufficient, or deficient. Data on age, gender, comorbidities, fracture type, and injury mechanism were collected and analyzed using IBM SPSS Statistics v26 (IBM Corp., Armonk, USA).

Results: A total of 116 elderly patients (mean age 75.32 ± 9.50 years; 66.4% women) with neck of femur fractures were enrolled. The majority (79.3%) had suboptimal vitamin D status, with 59.5% having insufficient and 19.8% having deficient levels. Only 20.7% had normal vitamin D levels. Advancing age (p = 0.003) and female gender (p = 0.002) showed significant associations with VDD. Trivial trauma, such as a slip or a fall, accounted for 87.9% of fractures, while the AO Foundation/Orthopedic Trauma Association (AO/OTA) classification revealed simple pertrochanteric (31A1) as the most frequent fracture type (46.6%). However, vitamin D status was not significantly associated with fracture classification (p = 0.573) or mode of injury (p = 0.414).

Conclusion: VDD is highly prevalent among elderly patients presenting with neck of femur fractures, particularly in older women. Advancing age and female gender are significant predictors of this deficiency. These findings emphasize the need for routine screening and timely correction of vitamin D levels as a preventive strategy against fragility fractures in the elderly population.

## Introduction

Neck of femur fractures are a major source of morbidity and mortality in the elderly (defined as ≥60 years, consistent with Indian and WHO guidelines), leading to functional decline, loss of independence, and increased healthcare costs. With the aging global population, the incidence of such fractures is rising and is expected to exceed six million annually by 2050 [1]. Over 90% of proximal femur fractures involve either the femoral neck or pertrochanteric regions, while subtrochanteric fractures account for the remaining 5% to 10% [2].

These fractures are assessed radiologically and classified according to the AO Foundation/Orthopedic Trauma Association (AO/OTA) classification system, which provides a standardized and widely accepted framework for proximal femoral fractures [3]. Several risk factors contribute to the development of neck of femur fractures, including advanced age, female gender, history of falls, medication use, smoking, alcohol intake, genetic predisposition, and nutritional deficiencies such as vitamin D deficiency (VDD) [4].

Osteoporosis is a key underlying condition in many of these cases. It renders bones porous, brittle, and fragile, significantly increasing the risk of fractures. Defined by the WHO as a disease marked by low bone mass and microarchitectural deterioration of bone tissue, osteoporosis leads to enhanced fragility and fracture susceptibility [5]. Non-modifiable risk factors include age and gender, while modifiable ones include VDD, smoking, low physical activity, and low BMI [6].

Vitamin D, primarily obtained through sunlight exposure and dietary sources like fish oils and mushrooms, plays a central role in bone health. However, a large portion of the Indian population is vegetarian, and combined with urban lifestyles characterized by indoor living, sunscreen use, and poor air quality, this leads to reduced cutaneous vitamin D synthesis. Consequently, VDD is highly prevalent across age groups and regions, with studies reporting deficiency rates ranging from 50% to 94.2% globally [7].

Vitamin D is essential for calcium and phosphate metabolism and bone mineralization. VDD impairs bone strength and increases fracture risk. Elderly individuals are particularly vulnerable due to factors such as reduced sun exposure, impaired skin synthesis, poor nutrition, and decreased hepatic and renal conversion of vitamin D [8].

VDD also contributes to muscle weakness and poor balance, heightening fall and fracture risks. Some studies suggest that VDD may delay fracture healing and increase mortality in hip fracture patients [9]. Identifying at-risk patients is crucial for implementing effective prevention strategies. Although vitamin D supplementation is shown to lower fracture risk, the optimal dosage and treatment duration remain under investigation [10].

Hip and proximal femur fractures in the elderly represent a growing public health concern due to their high morbidity, mortality, and socioeconomic burden. VDD, a modifiable risk factor, is highly prevalent in the Indian population, yet its role in fragility fractures is underreported in this region. Most available literature originates from Western countries, where differences in dietary habits, sun exposure, and genetic factors may influence vitamin D status and fracture risk. Limited Indian data exist on the association between serum vitamin D levels and fracture patterns in elderly patients. Understanding this relationship in our population is crucial to guide preventive strategies such as early screening, supplementation, and fall-prevention programs, thereby potentially reducing fracture incidence and improving outcomes. Therefore, this present cross-sectional observational study was conducted to assess the association between serum vitamin D levels and neck of femur fractures in elderly patients and identify demographic and clinical variables influencing this relationship.

## Materials and methods

Study setting and duration

This cross-sectional observational study was conducted over a duration of 18 months, from October 1, 2023, to March 31, 2025. Ethical approval for the study was obtained from the Ethics Committee of the Himalayan Institute of Medical Sciences, Swami Rama Himalayan University, Dehradun, Uttarakhand, India (approval number: SHRU/HIMS/ETHICS/2025/83) prior to initiation. Elderly patients presenting with neck of femur fractures were recruited after obtaining informed written consent. The study aimed to explore the relationship between serum vitamin D levels and neck of femur fractures in the population.

Sample size and sampling method

The minimum sample size was calculated to be 92 based on an estimated 60% prevalence of VDD in the elderly population. The formula used for sample size calculation was: 

$n = \frac{Z^2_{(1-\alpha/2)} \times P(1 - P)}{L^2}$,

where Z = 1.96 (at 95% confidence), P = 0.6, and L = 10% absolute precision. Despite the minimum requirement, a total of 116 patients were successfully enrolled using consecutive sampling.

Inclusion and exclusion criteria

Elderly patients (defined as ≥60 years of age, consistent with Indian and WHO guidelines) presenting with a neck of femur fracture were included in the study. Exclusion criteria included a history of pathological fractures, previous hip surgery, chronic hepatic or renal disease, and the use of medications that affect bone metabolism, such as phenytoin or corticosteroids. Patients with current or recent (past six months) vitamin D or calcium supplementation were excluded to avoid confounding.

Study tools and measurements

Serum 25-hydroxyvitamin D (25(OH)D) levels were assessed using a standardized enzyme-linked immunosorbent assay (ELISA) technique. Based on the results, vitamin D status was categorized into four groups: levels between 75 and 250 nmol/L were considered normal, levels ranging from 25 to 74 nmol/L were classified as insufficient, levels below 25 nmol/L were considered deficient, and levels exceeding 250 nmol/L were regarded as toxic. Pelvic X-rays in anteroposterior and standard lateral views were taken to assess and classify the type of fracture according to the AO/OTA classification system. A semi-structured case recording form was used to collect demographic, clinical, and laboratory information.

Data collection procedure

Patients who fulfilled the inclusion criteria underwent a general physical examination and were interviewed using a semi-structured format. For each patient, demographic details such as age, gender, and religion were recorded. Clinical information, including comorbidities, history of medication use, and any prior fractures, was documented. Laboratory investigations included serum vitamin D levels, kidney function tests (KFTs), and complete blood count (CBC). Radiological assessment was performed using X-rays, which were reviewed to determine the type and severity of the fracture.

Statistical analysis

Data were entered in Microsoft Excel (Microsoft Corp., Redmond, USA) and analyzed using IBM SPSS Statistics v26 (IBM Corp., Armonk, USA). Qualitative variables were summarized using frequencies, while quantitative variables were described using appropriate descriptive statistics. Categorical variables were analyzed using the chi-square test or Fisher’s exact test, with a p-value <0.05 considered statistically significant.

## Results

The mean age of the study population was 75.32 ± 9.50 years. Most participants belonged to the age group 71-80 years (37.9%), followed by 60-70 years (33.6%). Patients aged 81-90 years (20.7%) and those above 90 years (7.8%) formed smaller subgroups. In terms of gender, female patients constituted the majority (66.4%), while male patients accounted for 33.6%. The majority of patients were Hindu (88.8%), while Muslims comprised 6.9%, Christians 3.4%, and Sikhs 0.9%. Regarding comorbidities, cardiac disease, including hypertension, congestive heart failure (CHF), or coronary artery disease (CAD), was the most common (37.1%), followed by diabetes (29.3%). Pulmonary diseases such as asthma, chronic obstructive pulmonary disease (COPD), or tuberculosis were reported in 7.8%, while other comorbidities, including cancer, psychological illnesses, thyroid disorders, or scleroderma, were observed in 6.9% (Table 1).

**Table 1:** Demographic and clinical characteristics of study participants. This table presents the baseline demographic and clinical profile of the patients enrolled in the study. It shows the distribution of age groups with mean age, gender composition, religious background, and the prevalence of comorbidities such as cardiac disease, diabetes, pulmonary disorders, and other systemic conditions. HTN: Hypertension; CHF: Congestive heart failure; CAD: Coronary artery disease; COPD: Chronic obstructive pulmonary disease; PTB: Pulmonary tuberculosis

Variable	Domain	N (%)
Age group	60-70 Yrs	39 (33.6%)
	71-80 Yrs	44 (37.9%)
	81-90 Yrs	24 (20.7%)
	>90 Yrs	9 (7.8%)
Gender	Male	39 (33.6%)
	Female	77 (66.4%)
Religion	Hindu	103 (88.8%)
	Muslim	8 (6.9%)
	Christian	4 (3.4%)
	Sikh	1 (0.9%)
Comorbidities	Cardiac disease (HTN, CHF, or CAD)	43 (37.1%)
	Diabetes	34 (29.3%)
	Pulmonary disease (asthma, COPD, or PTB)	9 (7.8%)
	Others (cancer, psychological illness, thyroid disorder, scleroderma)	8 (6.9%)

The majority of fractures occurred due to trivial trauma, such as a slip and fall or a fall from bed/chair, accounting for 87.9% of cases. Falls from height were responsible for 5.2%, while road traffic accidents and other causes, such as animal hits or assaults, accounted for 6.9%. Based on the AO/OTA classification, the most common fracture type was simple pertrochanteric (31A1), observed in 46.6% of patients. Multifragmentary pertrochanteric (31A2) fractures were seen in 25%, while subcapital (31B1) fractures accounted for 17.2%. Less common were transcervical (31B2) fractures at 6% and basicervical (31B3) fractures at 5.2% (Table 2).

**Table 2:** Distribution of study participants based on mode of injury and AO/OTA fracture classification. This table summarizes the mechanism of injury leading to neck of femur fractures, highlighting the predominance of trivial trauma such as slips and falls, along with less frequent causes, including falls from height and road traffic accidents. It also details the fracture types according to the AO/OTA classification system, with proportions of simple pertrochanteric, multifragmentary pertrochanteric, subcapital, transcervical, and basicervical fractures. AO/OTA: AO Foundation/Orthopedic Trauma Association; RTA: Road traffic accident

Variable	Domain	N (%)
Mode of injury	Trivial trauma (slip and fall, fall from bed or chair)	102 (87.9%)
	Fall from height (fall from roof or stairs)	6 (5.2%)
	RTA and others (hit by animal or assault)	8 (6.9%)
AO/OTA classification	31A1: Simple pertrochanteric	54 (46.6%)
	31A2: Multifragmentary pertrochanteric	29 (25%)
	31B1: Subcapital	20 (17.2%)
	31B2: Transcervical	7 (6%)
	31B3: Basicervical	6 (5.2%)

The mean serum vitamin D level among the study participants was 52.93 ± 31.32 nmol/L. A majority had suboptimal vitamin D status, with 59.5% having insufficient and 19.8% having deficient levels. Only 20.7% of patients had normal vitamin D levels (Table 3).

**Table 3:** Distribution of vitamin D status among study participants. This table provides the distribution of serum vitamin D levels in the study population. It includes the mean vitamin D value and categorizes participants into normal, insufficient, and deficient groups, demonstrating the high prevalence of suboptimal vitamin D status. SD: Standard deviation

Vitamin D status	N (%)
Mean vitamin D level (± SD)	52.93 ± 31.32 nmol/L
Normal	24 (20.7%)
Insufficient	69 (59.5%)
Deficient	23 (19.8%)

A significant association was observed between age groups and vitamin D status (p = 0.003). Among patients aged 60-70 years, 47.8% were vitamin D deficient, 36.2% had insufficient vitamin D, and 12.5% had normal levels. In the 71-80 years group, deficiency was 13%, insufficiency was 37.7%, and 62.5% had normal levels. In the 81-90 years group, 34.8% were deficient, 14.5% had insufficient vitamin D, and 25% had normal levels. Among those above 90 years, deficiency was 4.3%, insufficiency was 11.6%, and none had normal vitamin D levels. Gender was also significantly associated with vitamin D status (p = 0.002). Among male patients, 4.3% were deficient, 44.9% had insufficient levels of vitamin D, while 29.2% had normal vitamin D levels. In contrast, female patients had a much higher prevalence of deficiency at 95.7%, with 55.1% having insufficient and 70.8% having normal levels. Religion showed no statistically significant association with vitamin D status (p = 0.307). Among Hindus, 78.3% were vitamin D deficient, 89.9% were vitamin D insufficient, and 95.8% had normal levels. Muslims had a 13% deficiency and 5.8% insufficiency, while 4.2% had normal levels. Christians had 4.3% deficiency and 4.3% insufficiency, but none had normal levels. Sikhs accounted for 4.3% of deficiency, while none were in the insufficient or normal categories (Table 4).

**Table 4:** Association between demographic variables and vitamin D status. This table shows the relationship between vitamin D status (deficient, insufficient, and normal) and demographic variables, including age group, gender, and religion. It presents the frequency distribution of vitamin D status within each subgroup and the corresponding p-values, indicating statistically significant associations (*) with age and gender.

Variable	Domain	Deficient	Insufficient	Normal	P-value	Chi-square
Age group	60-70 Yrs	11 (47.8%)	25 (36.2%)	3 (12.5%)	0.003*	19.50
	71-80 Yrs	3 (13%)	26 (37.7%)	15 (62.5%)		
	81-90 Yrs	8 (34.8%)	10 (14.5%)	6 (25%)		
	>90 Yrs	1 (4.3%)	8 (11.6%)	0		
Gender	Male	1 (4.3%)	31 (44.9%)	7 (29.2%)	0.002*	13.00
	Female	22 (95.7%)	38 (55.1%)	17 (70.8%)		
Religion	Hindu	18 (78.3%)	62 (89.9%)	23 (95.8%)	0.307	7.15
	Muslim	3 (13%)	4 (5.8%)	1 (4.2%)		
	Christian	1 (4.3%)	3 (4.3%)	0		
	Sikh	1 (4.3%)	0	0		

There was no statistically significant association between vitamin D status and mode of injury (p = 0.414). Among patients with trivial trauma, such as a slip and fall or a fall from a bed/chair, 91.3% were vitamin D deficient, 85.5% had insufficient vitamin D, and 91.7% had normal levels. For those with a fall from height, 8.7% were deficient in vitamin D, 5.8% had insufficient levels, and none had normal levels. In road traffic accidents and other injuries, none were vitamin D deficient; however, 8.7% had insufficient levels and 8.3% had normal levels. Similarly, no significant association was found between vitamin D status and AO/OTA fracture classification (p = 0.573). The most common fracture type was simple pertrochanteric (31A1), accounting for 56.5% of deficient cases, 42% of insufficient cases, and 50% of normal cases. Multifragmentary pertrochanteric (31A2) fractures comprised 21.7% of deficiency, 24.6% of insufficiency, and 29.2% of normal cases. Subcapital fractures (31B1) were observed in 17.4% of deficiency, 21.7% of insufficiency, and 4.2% of normal cases. Transcervical fractures (31B2) were not found in deficiency cases but occurred in 7.2% of insufficiency and 8.3% of normal cases. Basicervical fractures (31B3) were the least common, seen in 4.3% of deficiency, 4.3% of insufficiency, and 8.3% of normal cases (Table 5).

**Table 5:** Association between mode of injury, AO/OTA fracture classification, and vitamin D status. This table depicts the distribution of vitamin D status across different mechanisms of injury and fracture types classified by AO/OTA. It highlights the predominance of trivial trauma across all vitamin D categories and compares the prevalence of deficiency, insufficiency, and normal levels among various fracture patterns. The p-values are provided to indicate statistical significance. AO/OTA: AO Foundation/Orthopedic Trauma Association; RTA: Road traffic accident

Variable	Domain	Deficient	Insufficient	Normal	P-value	Chi-square
Mode of injury	Trivial trauma (slip and fall, fall from bed or chair)	21 (91.3%)	59 (85.5%)	22 (91.7%)	0.414	3.94
	Fall from height (fall from roof or stairs)	2 (8.7%)	4 (5.8%)	0		
	RTA and others (hit by animal or assault)	0	6 (8.7%)	2 (8.3%)		
AO/OTA classification	31A1: Simple pertrochanteric	13 (56.5%)	29 (42%)	12 (50%)	0.573	6.66
	31A2: Multifragmentary pertrochanteric	5 (21.7%)	17 (24.6%)	7 (29.2%)		
	31B1: Subcapital	4 (17.4%)	15 (21.7%)	1 (4.2%)		
	31B2: Transcervical	0	5 (7.2%)	2 (8.3%)		
	31B3: Basicervical	1 (4.3%)	3 (4.3%)	2 (8.3%)		

## Discussion

The present study was conducted to evaluate the association between serum vitamin D levels and neck of femur fractures (both intracapsular and extracapsular) among elderly patients at a tertiary care center in northern India. Among the 116 enrolled patients, a striking 79.3% (n = 92) had subnormal vitamin D levels, with 59.5% (n = 69) having insufficient and 19.8% (n = 23) having deficient levels. The mean serum vitamin D level was 52.93 ± 31.32 nmol/L, highlighting a high prevalence of hypovitaminosis D in this population.

Our findings are consistent with several previous studies. Han et al. [7] reported a severe deficiency in 83.2% of hip fracture patients and insufficiency in 11.9%, with only 4.9% having normal levels. Similarly, Khadgawat et al. [11] observed near-universal deficiency, with a mean level of 9.9 ± 4.8 ng/mL and 96.7% of patients showing VDD. Fatma et al. [12] and Ramesh et al. [13] also documented a high prevalence of suboptimal vitamin D levels among elderly fracture patients. These findings collectively support the notion that VDD is a widespread concern among elderly individuals with hip fractures and reinforce its role as a contributing factor to impaired bone health and increased fracture risk.

In our study, age was significantly associated with vitamin D status (p = 0.003), with the highest deficiency rates observed in patients aged 81-90 years. This is biologically plausible given the age-related decline in cutaneous vitamin D synthesis, reduced dietary intake, and limited sun exposure. Similar age associations were noted by Fatma et al. [12], although some studies, such as those by Han et al. [7], Singh et al. [14], and Hublikar et al. [9], did not find statistically significant age-based differences, suggesting possible regional or lifestyle influences.

Gender differences were also apparent. Female patients made up 66.4% (n = 77) of our sample and had significantly higher rates of VDD compared to male patients (p = 0.002). This finding aligns with studies by Han et al. [7], Vellingiri et al. [15], and Fatma et al. [12], all of which reported a female predominance and higher deficiency rates, likely due to postmenopausal hormonal changes, lower bone mass, and reduced sun exposure. Singh et al. [14], while reporting equal gender distribution, also noted lower vitamin D levels in female patients, although without statistical significance.

The majority of patients (88.8%, n = 103) in our study identified as Hindu, with Muslims, Christians, and Sikhs making up the rest. While Hindu patients showed a greater prevalence of VDD, the association with religion was not statistically significant (p > 0.05). These patterns mirror findings from Singh [16] and may reflect varying cultural practices, dietary habits, or patterns of sun exposure, though religion itself does not appear to be an independent risk factor.

Regarding injury mechanisms, most fractures (87.9%, n = 102) were caused by trivial trauma such as falls from standing height - typical of osteoporotic fractures. VDD was more prevalent in these low-energy trauma patients. Fatma et al. [12] similarly found that 86% of fractures were due to falls, and Khadgawat et al. [11] reported that 76.7% of fractures occurred at home due to slipping. In contrast, studies like Garg et al. [17] documented higher rates of road traffic accident-induced fractures, indicating potential demographic and environmental differences.

Comorbidities such as cardiovascular disease (37.1%, n = 43) and diabetes (29.3%, n = 34) were common in our cohort. Although not statistically analyzed in detail, comorbid conditions likely contribute to impaired vitamin D metabolism or reduced mobility and sun exposure. Fatma et al. [12] and Singh [16] also identified similar patterns, suggesting systemic health issues play a contributory role in both VDD and fracture risk.

Fracture classification based on AO/OTA revealed simple pertrochanteric fractures as the most common type (46.6%, n = 54), followed by multifragmentary pertrochanteric (25%, n = 29) and subcapital fractures (17.2%, n = 20). Although patients with extracapsular fractures appeared to have higher VDD, the association was not statistically significant (p > 0.05). Han et al. [7] found a significant relationship between extracapsular fractures and VDD, while Fatma et al. [12] and Hublikar et al. [9] also showed similar correlations. Differences in fracture classification and small subgroup sizes may account for inconsistencies in findings.

The overall high prevalence of VDD in this study population underscores the importance of routine screening for vitamin D, especially in elderly individuals at risk of falls and fractures. As a modifiable risk factor, early detection and correction of VDD could potentially reduce fracture incidence and improve outcomes in this vulnerable population.

The strengths of this study lie in its cross-sectional observational design, standardized biochemical assessment of vitamin D, and systematic fracture classification. However, being a single-center study with a relatively small sample size, the findings may not be generalizable to wider populations. The absence of long-term follow-up and interventional assessment of supplementation further limits conclusions regarding causal relationships and outcomes. Future research should focus on multicentric, large-scale studies and randomized controlled trials to better establish the role of vitamin D in fracture prevention, bone healing, and long-term functional outcomes.

## Conclusions

This study highlights the high prevalence of VDD among elderly individuals with neck of femur fractures, especially in women and advanced age groups. As a modifiable risk factor, early detection and correction of VDD should be incorporated into geriatric fracture prevention programs. Clinicians should consider routine screening and supplementation in at-risk populations to reduce fracture incidence and improve outcomes. Future multicentric studies with larger sample sizes and longitudinal follow-up are recommended to validate these findings.
